# PReS-FINAL-2255: Primary pyomiositis in children: a challenging diagnosis

**DOI:** 10.1186/1546-0096-11-S2-P245

**Published:** 2013-12-05

**Authors:** V Moressa, S Pastore, M Pavan, C Bracaglia, L Lepore

**Affiliations:** 1 IRCCS Burlo Garofolo, Trieste, Italy; 2 Bambino Gesù Paediatric Hospital, Rome, Italy

## Introduction

Primary Pyomiositis (PM) is an uncommon and potentially serious bacterial infection of striated muscle. It is typically a tropical disease but its frequency has been increasing in the Western Countries.

*Staphylococcus Aureus *is the most frequently isolated organism from blood samples of patients affected by this condition. The rarity in temperate climates of this potentially fatal disease and its non-specific signs can represent a diagnostic challenge, leading to a dangerous diagnostic delay.

## Objectives

The aim of our study is to identify a common diagnostic profile in order to provide an early diagnosis.

## Methods

A retrospective review was conducted to analyze the experience of two pediatric Italian hospitals (Clinica Pediatrica, IRCCS Burlo Garofolo, Trieste and Ospedale Pediatrico Bambino Gesù, Roma) from 2005 to 2013.

## Results

Over the 8-year period, 12 cases were identified (8 boys and 4 girls). None of them presented immunocompromising conditions.

Mean age at diagnosis was 10,8 years (range 3 months-15 years); the most commonly involved muscle was the gluteal one (8/12 cases); in 4/12 cases other muscles were involved (iliopsoas, obturator and abdominal oblique muscle).

All of the patients presented with pain, fever and limp with limitation in movements.

Physical examination revealed in all cases limitation on flexion and abduction of the hip and pain at pressure of the involved muscles.

One patient, a 3 months-old baby, presented with fever, irritability, lack of appetite and pain at the attempt to move the affected muscles.

A history of local trauma was present in two cases. Laborator findings showed rise in acute phase reactants in all cases, whereas creatinine kinase was normal in all our patients. Xray and Ultrasound examinations were performed in all cases and were always negative. On the contrary MRI was diagnostic in all cases, showing swelling of the involved muscles.

In all cases antibiotic therapy was conducted intravenously for two weeks, therefore orally for a total of four-six weeks. One patient required surgical drain.

## Conclusion

PM is a rare deep, subacute bacterial infection of the skeletal muscle.

Once considered a tropical countries' prerogative, now it is appearing more frequently in temperate climate areas.

Its rarity and the lack of specific symptoms can lead to a diagnostic delay and fatal complications. Septic arthritis represents the main differential diagnosis, therefore MRI, the gold standard for this disease, should be performed as soon as possible in any child with fever, pain and limp without a precise articular involvement and a recognized infectious focus.

## Disclosure of interest

None declared.

